# Visual-Inertial RGB-D SLAM with Encoder Integration of ORB Triangulation and Depth Measurement Uncertainties

**DOI:** 10.3390/s24185964

**Published:** 2024-09-14

**Authors:** Zhan-Wu Ma, Wan-Sheng Cheng

**Affiliations:** School of Electronic and Information Engineering, University of Science and Technology Liaoning, Anshan 114051, China

**Keywords:** covariance intersection filter, encoders, RGB-D SLAM, multisensor fusion

## Abstract

In recent years, the accuracy of visual SLAM (Simultaneous Localization and Mapping) technology has seen significant improvements, making it a prominent area of research. However, within the current RGB-D SLAM systems, the estimation of 3D positions of feature points primarily relies on direct measurements from RGB-D depth cameras, which inherently contain measurement errors. Moreover, the potential of triangulation-based estimation for ORB (Oriented FAST and Rotated BRIEF) feature points remains underutilized. To address the singularity of measurement data, this paper proposes the integration of the ORB features, triangulation uncertainty estimation and depth measurements uncertainty estimation, for 3D positions of feature points. This integration is achieved using a CI (Covariance Intersection) filter, referred to as the CI-TEDM (Triangulation Estimates and Depth Measurements) method. Vision-based SLAM systems face significant challenges, particularly in environments, such as long straight corridors, weakly textured scenes, or during rapid motion, where tracking failures are common. To enhance the stability of visual SLAM, this paper introduces an improved CI-TEDM method by incorporating wheel encoder data. The mathematical model of the encoder is proposed, and detailed derivations of the encoder pre-integration model and error model are provided. Building on these improvements, we propose a novel tightly coupled visual-inertial RGB-D SLAM with encoder integration of ORB triangulation and depth measurement uncertainties. Validation on open-source datasets and real-world environments demonstrates that the proposed improvements significantly enhance the robustness of real-time state estimation and localization accuracy for intelligent vehicles in challenging environments.

## 1. Introduction

In recent years, with the rapid development of robotics, computer vision, autonomous driving, augmented reality/virtual reality, planetary exploration, and unmanned aerial vehicle navigation [1], visual odometry and SLAM have emerged as prominent areas of research [2]. SLAM technology enables robots to not only achieve real-time self-localization but also to construct and continuously update maps of unknown environments during exploration, significantly enhancing their ability to operate autonomously in such settings [3]. In the field of robotics, intelligent wheeled robots have emerged as a prominent area of research [4]. Based on the types of sensors employed, mainstream SLAM systems are primarily classified into laser-based SLAM, vision-based SLAM, and various sensor-assisted laser/vision multisensor fusion SLAM technologies. Visual SLAM relies on cameras as the primary sensors, and its ability to capture rich information, combined with its lightweight and cost-effective nature, has garnered significant interest from researchers [5]. Within the domain of visual SLAM, ORB-SLAM3 demonstrates superior performance, surpassing other algorithms in terms of accuracy, computational efficiency, and robustness [6]. Currently, ORB-SLAM3 acquires the 3D positions of feature points by directly utilizing RGB-D depth camera measurements, without accounting for the uncertainty in triangulation estimation. Furthermore, ORB-SLAM3 fuses data from the RGB-D depth camera and the IMU, but it does not integrate wheel encoder data, which are crucial for improving SLAM accuracy and aiding mobile robot localization and navigation. The literature [7] proposes a lightweight multisensor fusion method involving ORB-SLAM, IMU, and wheel odometry for the localization and navigation of an indoor mobile robot in a GPS-denied environment. Experimental results demonstrate that the robot can localize itself with tolerable error and exhibit strong navigation capabilities in specific scenarios. The ORB-SLAM in this study employs a monocular camera to compute the real-time camera position through feature matching. To filter out dynamic objects during localization and mapping, the literature [8] presents a mobile robot localization method using asynchronous data fusion comprising a wheel odometer and a visual odometer within an Extended Kalman Filter (EKF) framework, based on a semantic SLAM system. The approach integrates semantic SLAM based on ORB-SLAM2 with YOLOv3. The asynchronous data fusion for mobile robot localization, comprising visual and wheel odometer data, is implemented at a frequency of approximately 50 Hz using EKF. Experimental results demonstrate that this method effectively improves the accuracy and robustness of the mobile robot. Recently, the literature [9] proposed a visual-inertial-wheel odometry method that provides robust initialization and highly accurate estimates for ground robots. This study utilizes a novel maximum-a-posteriori initialization, coupled with wheel encoder measurements, to address the unobservable scale problem in visual-inertial-only initialization during straight-line motion at the beginning of the trajectory. Experiments on public datasets demonstrate the effectiveness and efficiency of the initialization, the robustness of pose tracking, and the improved accuracy of the entire trajectory. In addition, the literature [10] utilizes a CI filter to fuse inertial navigation sensors and wheel odometry to improve the accuracy of real-time localization and mapping. The effectiveness and feasibility of the algorithm are validated using an experimental platform. The aforementioned literature demonstrates that the use of wheel encoders not only enhances the accuracy and robustness of SLAM but also addresses the initialization failures in mobile robots. Inspired by this literature, this paper proposes a novel tightly coupled visual-inertial RGB-D SLAM with encoder integration of ORB triangulation and depth measurement uncertainties to improve ORB-SLAM3.

Visual odometry (VO) algorithms can be categorized into two main types: feature-based methods and direct methods [11]. However, in both feature-based and direct methods within SLAM, accurately estimating the uncertainty of the camera pose and the 3D positions of feature points is crucial. Such accurate estimations are essential for selecting precise keyframes, particularly in the context of information fusion and active SLAM [12,13]. The primary objective is to minimize uncertainty or entropy, which necessitates a closed-form solution for the uncertainty estimation of the camera pose and the 3D positions of feature points. The uncertainty estimation of the camera pose [1] and the 3D positions of feature points are key parameters for SLAM. This uncertainty can generally be expressed using the covariance matrix [14], information entropy [15], and Fisher’s information matrix [13]. The estimations of uncertainty in the camera poses and the 3D positions of feature points are primarily based on filters (e.g., the Extended Kalman Filter) [16] and nonlinear optimization methods (e.g., Bundle Adjustment and graph optimization) [17]. Uncertainty estimations based on filter methods are both convenient and fast, providing direct access to the covariance matrix or expected entropy. In contrast, nonlinear optimization-based methods do not directly estimate uncertainty. As a result, the covariance matrix and information entropy are not optimized as direct parameters. Instead, uncertainty in nonlinear optimization requires rigorous computation [18].

In the study of Vakhitov et al. [19], a PnP(L) solver based on EPnP and DLS is proposed for uncertainty-aware pose estimation by considering both the 3D coordinates and 2D projections of feature points in SLAM. Additionally, the motion-only bundle adjustment is modified to account for the uncertainty in the 3D positions of feature points. Tests performed on the KITTI datasets demonstrate improved accuracy. In Belter et al. (2016) [20], two spatial uncertainty models are proposed, based on experimental tests, to estimate the covariance matrix of the measured feature points. The resulting covariance is then incorporated into factor graph optimization in the back-end of the SLAM system. While these methods provide error models and uncertainties for 3D points, they cannot be directly applied to more general visual SLAM systems. In Belter et al. (2018) [21], the problem of estimating the uncertainty in the 3D positions of feature points using the feature method is discussed in detail. The effect of point feature uncertainty on trajectory and map uncertainty estimation is investigated through an uncertainty model. A factor graph optimization model is employed to minimize the error in the 3D positions of feature points measured by depth cameras. Experiments conducted on open-source datasets demonstrate that the algorithm can improve the accuracy of camera pose estimation.

Currently, researchers mainly focus on one type of uncertainty study, such as the 3D positions of feature points [22,23,24] or camera poses [25,26,27,28]. Li and Yang [29] present a pose fusion method that accounts for the possible correlations among measurements. The handling of these correlations is based on the theory of Covariance Intersection (CI), where the independent and dependent parts are separated to yield a more consistent result. Few studies have investigated the problem of joint uncertainty estimation for the 3D positions of feature points and camera pose. However, the above methods are unable to provide closed-form solutions for uncertainty estimations, which are crucial for information fusion and active SLAM, as motion prediction and pose evaluation require one or even multiple steps of forward-looking uncertainty propagation. To address the above problems, this paper proposes a closed-form uncertainty estimation algorithm, called CI-TEDM (Covariance Intersection for Triangulation Estimates and Depth Measurements), for fusing uncertainties in triangulation estimates and depth measurements of the 3D positions of feature points. This algorithm is based on the closed-form uncertainty estimation of both camera poses and 3D positions of feature points. On one hand, based on the ORB-SLAM3 system, the covariance matrix of the camera pose and the triangulation of ORB features is estimated separately. On the other hand, the covariance matrix of the 3D positions of feature points obtained from the depth measurements of the RGB-D camera is computed. The 3D positions of feature points estimated by the two approaches are then fused with a Covariance Intersection (CI) filter, which uses the two covariance matrices as weights to achieve optimal fusion [18].

Moreover, the initialization of monocular SLAM can be time-consuming when the system lacks substantial disparity. Binocular SLAM requires more time to process stereo pairs [30]. Therefore, in this paper, RGB-D SLAM is used to achieve robustness and real-time performance. The open-source ORB-SLAM3 system with monocular, stereo, and RGB-D cameras has attracted much attention from scholars for its high accuracy and real-time performance [6]. Inspired by the IMU pre-integration model and the powerful and fast loop closure detection in [31], this paper introduces wheel encoder edges, which optimize the state of each frame to avoid tracking failures in RGB-D cameras and help improve the robustness of the entire RGB-D SLAM system [32]. Although RGB-D cameras can achieve centimeter-level or even higher accuracy, vision SLAM still faces significant challenges in complex environments, such as changes in illumination, moving objects, adverse weather, weak textures, and environmental degradation [33]. Cameras are prone to losing tracking information, which can lead to tracking failures due to fast camera motion, unstable performance of embedded boards, and low frame rates during image capture [34]. In this study, auxiliary sensors, such as IMU and encoders, are integrated to mitigate the problem of temporary tracking failures that may occur in pure-vision SLAM. Visual-inertial SLAM is a current research hotspot and can be categorized into two types: loosely coupled and tightly coupled [35]. Tightly coupled SLAM systems include direct methods [36], keyframe-based visual-inertial SLAM (OKVIS) [37], robust visual-inertial odometry based on EKF [38], and the monocular visual-inertial system (VINS-Mono) [39]. Among these methods, the most accurate results for the EuRoC datasets [40] are achieved by visual-inertial ORB-SLAM3, which is primarily based on the theoretical framework of real-time IMU pre-integration [6]. In this study, encoder and IMU data are fully utilized to enhance the robustness and real-time performance of RGB-D ORB-SLAM3.

The main contribution of this paper lies in the new combination of CI-TEDM and wheel odometry, which seeks to improve the accuracy and robustness of SLAM. Additionally, this paper derives the wheel encoder pre-integration model, error model, rotation-to-state-variable Jacobian matrix, and position-to-state-variable Jacobian matrix, which are used in local BA optimization to enhance SLAM results.

The rest of this paper is organized as follows. Section 2 outlines the framework of the system. Section 3 describes the fusion of ORB-SLAM3 triangulation estimation and depth measurement estimation algorithms. Section 4 derives the encoder pre-integration model, which provides the Jacobian matrix of the rotation-to-state variable and the position-to-state variable to facilitate local BA optimization for improved SLAM results. Section 5 validates the algorithms using open-source datasets and real-world environments, presenting the experimental results. Section 6 concludes the paper.

## 2. System Overview

**Figure 1:**
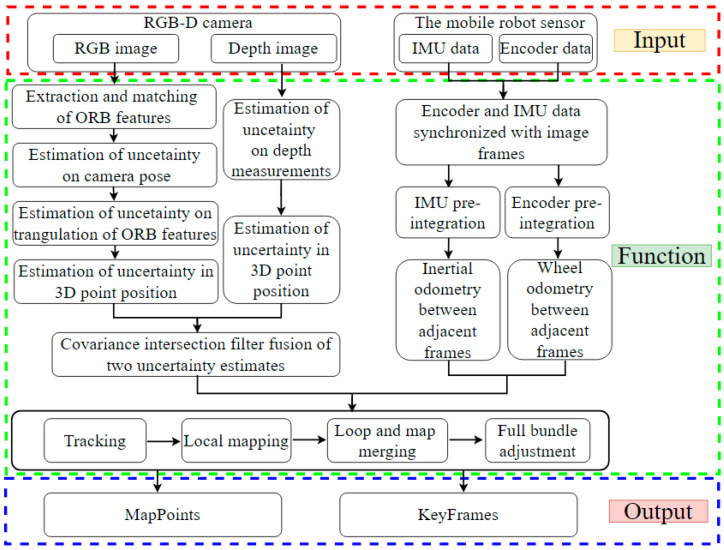
The system framework diagram. The system framework diagram consists of three main modules: input, function, and output.

The system framework diagram is shown in Figure 1, which consists of the input, function and output of the three main modules. The primary enhancement in the input module is the addition of a new encoder data thread. The system inputs include ORB images and RGB-D images from the RGB-D camera, encoder data from the wheel encoder, and IMU data from the IMU. In the function module, ORB feature points are first extracted and matched from each RGB image. The uncertainty in the camera pose is then derived using these matched point pairs. Given the observation noise of feature points in the RGB image plane, the uncertainty in the 6D camera pose ξ, represented by an element of the Lie algebra se(3), is estimated through implicit differentiation and covariance propagation. Utilizing the resulting camera pose uncertainty, the uncertainty in the triangulation of ORB feature points is computed, leading to the estimation of the uncertainty in the position of each 3D point. Simultaneously, the uncertainty in the depth measurements from the RGB-D camera is propagated to determine the uncertainty in the position of each 3D point. The uncertainties from both triangulation and depth measurements are then fused using a CI filter. Additionally, it is required to synchronize the encoder and IMU data with image frames within a permissible time alignment error. Encoder or IMU data that exceed the time alignment threshold relative to the nearest keyframe are discarded. The encoder and IMU data are then pre-integrated to compute the wheel and inertial odometry between adjacent image frames. The system with CI-TEDM that fuses IMU and encoder data based on ORB-SLAM3 is referred to as VIEOS3-TEDM, the system fusing only encoder data is termed VEOS3-TEDM, and the system using CI-TEDM alone is called VOS3-TEDM. An important modification is the addition of pure encoder edges in VEOS3-TEDM, which connect two states, $x_{i}$ and $x_{j}$, using only encoder measurements and are incorporated into the spanning tree. This tree primarily propagates corrected poses to all keyframes in the map. Finally, the CI filter fusion results and odometry results are input into the tracking, local mapping, loop closure, map merging, and full bundle adjustment (BA) modules for processing. In the output module, the map points of the environment and the keyframes of the mobile robot’s trajectory are obtained through the aforementioned processing.

## 3. Fusion of ORB-SLAM3 Triangulation and Depth Measurement Uncertainty Estimations

### 3.1. Uncertainty Estimation in ORB-SLAM3 Triangulation and Depth Measurement

**Figure 2:**
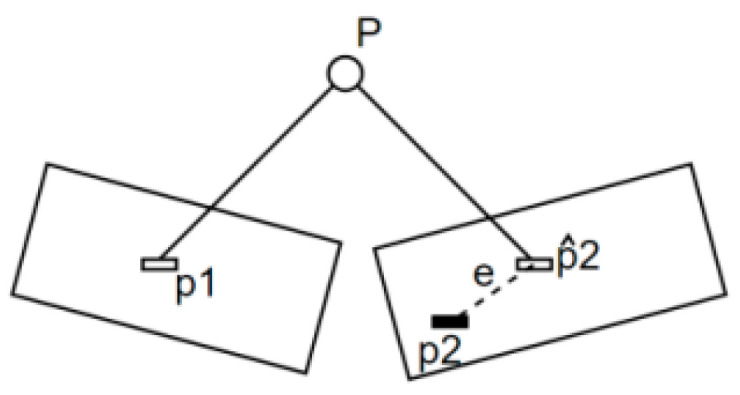
An example diagram of reprojection error. Feature matching indicates that points p1 and p2 are projections of the same spatial point p, but the camera pose is initially unknown. Initially, there is a certain distance between the projected point, $\overset{\wedge }{p}2$, of P and the actual point, p2. The camera pose is then adjusted to minimize this distance.

The first step is to estimate the uncertainty of the camera pose. Minimizing the reprojection error of 3D feature points is commonly used to estimate the camera pose, as illustrated in Figure 2. Once the uncertainty of the camera pose is determined, the uncertainty of the 3D feature points can be calculated. Simultaneously, the depth measurement uncertainty is also estimated. The derivation of the solution formula mentioned above can be found in Yuan et al. [18].

### 3.2. Fusion of Two Uncertainty Estimations

It is assumed that the two uncertainty estimates of the 3D feature points obtained using the camera follow a normal distribution, as denoted in Yuan et al. [18].

Np∧i,Trw,covp∧i,Trw and Np∧i,Depw,covp∧i,Depw,

where p∧i,Trw and covp∧i,Trw represent the uncertainty concerning the world coordinate system (denoted as w) and the covariance matrix of the 3D feature points triangulated by the camera, respectively. These estimates are then fused to obtain a high-precision map. According to the derivation formula for triangulation and depth measurement uncertainty estimation, it is evident that a correlation exists between the two normal distributions. In this paper, a CI filter is employed to fuse these two correlated estimates, which allows for the fusion of two estimates with unknown cross-covariance and ensures consistent results. The formula [18] is as follows:

(1) covp∧iw=ωcov−1p∧i,Trw+1−ω⋅cov−1p∧i,Depw−1

(2) p∧iw=covp∧iw⋅ωcov−1p∧i,Trww⋅p∧i,Tr+1−ω⋅cov−1p∧i,Depww⋅p∧i,Dep

where $\omega \in \left[0,1\right]$ is the weight assigned to the uncertainty in the triangulation estimate and the depth measurement estimate, which can be calculated by minimizing the trace of the fused covariance [18], i.e.,

(3) ω∗=argminωtrcovp∧iw .

## 4. Derivation of the Wheel Encoder Model

### 4.1. Pre-Integration Model for Wheeled Encoder

**Figure 3:**
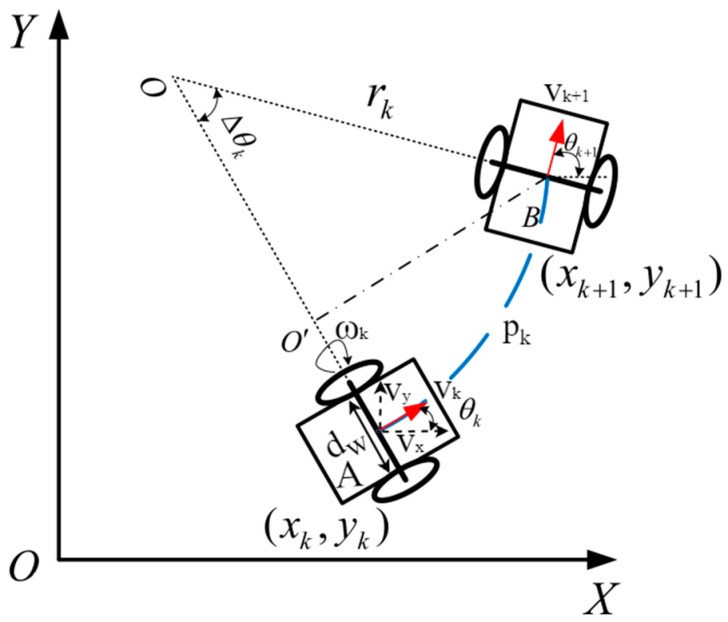
The motion model of the wheeled robot using wheel encoders. The figure illustrates the motion model of a mobile robot using wheel encoders in a 2D plane. The model describes the robot’s trajectory between its position at time $t_{k}$, denoted as $\left(x_{k+1},y_{k+1}\right)$, and its position at time $t_{k+1}$, denoted as $\left(x_{k+1},y_{k+1}\right)$.

There are multiple wheel encoder measurements between two adjacent frames [8]. In this paper, inspired by IMU pre-integration [6], a wheel encoder pre-integration model is derived to compute a low-frequency integration term that provides motion constraints for the two frames. Since the object of study is a ground mobile robot, its motion is approximated to be in a plane. Thus, the motion of the robot can be represented by a rotation angle, θ∈R, around the *z*-axis and a 2D translation vector, $q\in R^{2}$, in the x−y plane. The wheel encoder provides the mobile robot with measured values for linear velocity, v, and angular velocity, ω [41]. It is assumed that the rear wheel of the mobile robot is aligned with the positive *x*-axis of the wheel encoder coordinate system and that the left side of the rear wheel is aligned with the positive *y*-axis. The wheel encoder coordinate system follows the right-hand rule, and the movement model for the wheel odometry is shown in Figure 3. Figure 3 shows the robot moving from coordinate $\left(x_{k},y_{k}\right)$ at time $t_{k}$ to the coordinate $\left(x_{k+1},y_{k+1}\right)$ at time $t_{k+1}$. The linear velocity of the mobile robot is measured by an incremental photoelectric encoder, which converts pulse data into the robot’s linear velocity equation:

(4) $$v=\frac{\pi d_{w}}{N\Delta t}n,$$

where $d_{w}$ is the diameter of the rear wheel of the robot, N is the number of encoders lines, Δt is the sampling time, and n is the number of pulses during the sampling time. The linear velocities of the robot’s drive wheels are calculated by Equation (4), and the true velocities of the left and right wheels are obtained by subtracting measurement noise from the observed values as follows [42]:

(5) $$v_{k}^{el}={\tilde{v}}_{k}^{el}-\eta_{k}^{eld},$$

(6) $$v_{k}^{er}={\tilde{v}}_{k}^{er}-\eta_{k}^{erd},$$

where ${\left[\begin{array}{cc} \eta_{k}^{eld} & \eta_{k}^{erd} \end{array}\right]}^{T}\in {\mathbb{R}}^{2}$ is the measurement noise caused by the wheel encoders, which is assumed to follow a zero-mean Gaussian distribution with covariance matrix ${\left[\begin{array}{cc} \eta_{k}^{eld} & \eta_{k}^{erd} \end{array}\right]}^{T}\in {\mathbb{R}}^{2}$. $v_{k}^{el}$ and $v_{k}^{er}$ are the estimated instantaneous linear velocities at moment $t_{k}$. ${\tilde{v}}_{k}^{el}\;$ and ${\tilde{v}}_{k}^{er}$ are the linear velocities converted from the measurements of the left and right wheel encoders at moment $t_{k}$. It is assumed that the robot operates in an ideal environment where the surface is flat and the wheels do not slip. The linear velocity of the wheels is denoted by $v_{k}^{e}={\left[\begin{array}{ccc} {\tilde{v}}_{k}^{ex} & 0 & 0 \end{array}\right]}^{T}-\eta_{k}^{mvd}$, where $\eta_{k}^{m\omega d}=\;\;{\left[\begin{array}{ccc} \eta_{k}^{m\omega x} & \eta_{k}^{m\omega y} & \eta_{k}^{m\omega z} \end{array}\right]}^{T}\in {\mathbb{R}}^{3}$ denotes the measurement noise vector in the direction of x,y,z for the linear velocity. The angular velocity of the wheels is denoted by $\omega_{k}^{e}={\left[\begin{array}{ccc} 0 & 0 & {\tilde{\omega }}_{k}^{ez} \end{array}\right]}^{T}-\eta_{k}^{m\omega d}$, where ${\tilde{\omega }}_{k}^{ez}$ is the angular velocity converted from the measured value of the wheel encoder at moment $t_{k}$. $\eta_{k}^{m\omega d}=\;\;{\left[\begin{array}{ccc} \eta_{k}^{m\omega x} & \eta_{k}^{m\omega y} & \eta_{k}^{m\omega z} \end{array}\right]}^{T}\in {\mathbb{R}}^{3}$ represents the measurement noise vector in the direction of x,y,z for the angular velocity.

Due to the high frequency of the wheel encoder, the state transition model between two adjacent frames of wheel encoder measurements can be described as:

(7) $$\theta_{k+1}=\theta_{k}+\omega_{k}\cdot \Delta t,$$

(8) $$q_{k+1}=q_{k}+R\left(\theta_{k}\right)\cdot \left[\begin{array}{l} \;\;\;0\\ v_{k}\cdot \Delta t \end{array}\right].$$

Here, the Euler integration method is used, and by calculating Equations (7) and (8) in recursive form, the wheel encoder pre-integration term between two adjacent frames can be obtained. Since the ground robot operates in a 2D x−y plane, the rotation matrix is expressed as:

(9) $$R\left(\theta_{k}\right)=\left[\begin{array}{l} \cos\theta_{k}\;\;\;\;\;-\sin\theta_{k}\\ \sin\theta_{k}\;\;\;\;\;\cos\theta_{k} \end{array}\right].$$

Due to noise in the wheel encoder measurements, the following Equation (10) is used to construct the measurement error model:

(10) $$\begin{array}{l} \overset{-}{\omega }=\omega +n_{\omega }\\ \overset{-}{v_{x}}=v_{x}+n_{x}\\ \overset{-}{v_{y}}=v_{y}+n_{y} \end{array}.$$

In Equation (10), $v_{x}$ and $v_{y}$ denote the forward and left lateral linear velocities of the mobile robot, respectively. Assuming the noise of ω, $v_{x}$, and $v_{y}$ is Gaussian white noise, with Gaussian distributions as follows: $n_{\omega }\sim N\left(0,\delta_{\omega }^{2}\right),\;n_{v_{x}}\sim N\left(0,\delta_{v_{x}}^{2}\right),\;n_{v_{y}}\sim N\left(0,\delta_{v_{y}}^{2}\right)$.

A linear recurrence relation for the state error of the pre-integrated term of the wheel encoder can be derived from Equations (7), (8) and (10):

(11) $$\left[\begin{array}{l} \delta \theta_{k+1}\\ \delta q_{k+1} \end{array}\right]=P_{k}\left[\begin{array}{l} \delta \theta_{k}\\ \delta q_{k} \end{array}\right]+H_{k}\left[\begin{array}{l} n_{\omega }\\ n_{v} \end{array}\right].$$

In Equation (11), $n_{v}$ denotes the measurement noise of $v_{x}$ and $v_{y}$. The Jacobian matrices $P_{k}$ and $H_{k}$ can be calculated from Equations (12) and (13), respectively:

(12) $$P_{k}=\left[\begin{array}{l} \;\;\;\;\;\;\;\;\;\;\;\;\;\;\;\;\;\;1\;\;\;\;\;\;\;\;\;\;\;\;\;\;\;\;\;\;\;\;\;0_{1\times 2}\;\;\;\\ R\left(\theta_{k}\right)\cdot J\cdot \left[\begin{array}{l} 0\\ v_{k}\cdot \Delta t \end{array}\right]\;\;\;\;\;\;\;\;\;I_{2\times 2} \end{array}\right]\;\;\;\;,$$

(13) $$H_{k}=\left[\begin{array}{l} \Delta t\;\;\;\;\;\;\;\;\;\;\;0_{1\times 2}\\ 0_{2\times 1}\;\;\;\;R\left(\theta_{k}\right)\cdot \Delta t \end{array}\right],$$

(14) $$J=\left[\begin{array}{l} 0\;\;\;\;\;\;-1\\ 1\;\;\;\;\;\;\;\;0 \end{array}\right].$$

After obtaining the linear transition equation for the state error at adjacent moments, the covariance matrix of the wheeled encoder pre-integration term between neighboring image frames can be calculated using the following Equation (15):

(15) $$Q_{k+1}=P_{k}Q_{k}P_{k}^{T}+H_{k}\sum_{n}H_{k}^{T},$$

where $Q_{k}$ is the covariance matrix of the state quantities $\theta_{k}$ and $q_{k}$; $\sum_{n}$ is the covariance matrix of the noise, which is calculated by the following Equation (16):

(16) $$\sum_{n}=\left[\begin{array}{l} \delta_{\omega }^{2}\;\;\;\;\;0\;\;\;\;\;0\\ 0\;\;\;\;\;\delta_{v_{x}}^{2}\;\;\;\;\;0\\ 0\;\;\;\;\;0\;\;\;\;\;\;\;\delta_{v_{y}}^{2} \end{array}\right].$$

### 4.2. Pre-Integration Error of Wheeled Encoder

Based on the derivation of wheel encoder pre-integration in Section 4.1, the pre-integration error for the wheel encoders is [42]:

(17) $$r_{\Delta \phi_{ij}^{e}}^{e}\overset{\Delta }{=}Log[{\left(△{\tilde{R}}_{ij}^{e}\right)}^{T}\Delta R_{ij}^{e}],$$

(18) $$r_{\Delta p_{ij}^{e}}^{e}\overset{\Delta }{=}\Delta p_{ij}^{e}-△{\tilde{p}}_{ij}^{e}.$$

In the above equation, $\Delta R_{ij}^{e}$ and $\Delta p_{ij}^{e}$ are the predicted values of the wheel encoders pre-integration, and $△{\tilde{R}}_{ij}^{e}$ and $△{\tilde{p}}_{ij}^{e}$ are the observed values of the wheel encoders pre-integration.

#### 4.2.1. Jacobian Matrix of Rotation to State Variables

Equation (17) represents the rotational error in the pre-integration of the wheel encoders, which does not involve $p_{wi}^{e}$ and $p_{wj}^{e}$. Therefore, the Jacobian matrix with respect to each of these state variables is 0, and its Jacobian matrix with respect to $\delta \phi_{i}^{e}$ and $\delta \phi_{j}^{e}$ is given by [42]:

(19) $$\frac{\partial r_{\Delta \phi_{ij}^{e}}^{e}}{\partial \delta \phi_{i}^{e}}=-J_{r}^{-1}(r_{\Delta \phi_{ij}^{e}}^{e}){\left(R_{wi}^{eT}R_{wj}^{e}R_{ce}\right)}^{T},$$

(20) $$\frac{\partial r_{\Delta \phi_{ij}^{e}}^{e}}{\partial \delta \phi_{j}^{e}}=J_{r}^{-1}(r_{\Delta \phi_{ij}^{e}}^{e})R_{ce}^{T}.$$

In the above equation, $R_{ce}$ is the rotation matrix from the wheel encoder coordinate system to the camera coordinate system, which can be obtained from the intelligent vehicle model built in ROS.

#### 4.2.2. Jacobian Matrix of Position to State Variables

Equation (18) represents the pre-integrated position error of the wheel encoders. Its Jacobian matrix with respect to $p_{wi}^{e},p_{wj}^{e},\delta \phi_{i}$ and $\delta \phi_{j}$ is given by [42]:

(21) $$\frac{\partial r_{\Delta p_{ij}^{e}}^{e}}{\partial \delta p_{wi}^{e}}=-R_{ce}^{T}R_{wi}^{eT},$$

(22) $$\frac{\partial r_{\Delta p_{ij}^{e}}^{e}}{\partial \delta p_{wj}^{e}}=R_{ce}^{eT}R_{wi}^{eT},$$

(23) $$\frac{\partial r_{\Delta p_{ij}^{e}}^{e}}{\partial \delta \phi_{i}^{e}}=R_{ce}^{T}{\left(R_{wi}^{eT}(R_{wj}^{e}p_{ce}+p_{wj}^{e}-p_{wi}^{e})\right)}^{\wedge },$$

(24) $$\frac{\partial r_{\Delta p_{ij}^{e}}^{e}}{\partial \delta \phi_{j}^{e}}=-R_{ce}^{T}R_{wi}^{eT}R_{wj}^{e}p_{ce}^{\wedge }.$$

In the above equation, $p_{ce}$ is the translation matrix from the wheel encoder coordinate system to the camera coordinate system, which can be obtained from the intelligent vehicle model built in ROS.

## 5. Experimental Analysis of the VEOS3-TEDM Algorithm

### 5.1. Experimental Analysis of Open-Source Datasets

The combination of the three datasets—RGB-D, IMU, and wheel encoders—is not currently available in some public datasets. To validate the effectiveness of the VEOS3-TEDM algorithm proposed in this paper, open-source datasets from the literature [42] are used. These datasets are applied to a differential wheeled robot equipped with a Kinect v2 RGB-D camera, two-wheel encoders, and an IMU sensor. The ground truth data for these datasets are provided via a total station, which generates the true trajectory of the robot at approximately 10 Hz by measuring the position of a prismatic reflector fixed to the robot, with an accuracy close to 1 mm. These datasets were recorded using a mobile robot on an experimental platform, with captured scenes including long straight corridors and laboratories. To validate the deviation of the estimated trajectories from the true trajectories, a public benchmarking tool [43] was used to evaluate the Absolute Trajectory Error (ATE), which reflects the SLAM accuracy by calculating the Root-Mean-Squared Error (RMSE) of the system output. For evaluating algorithm accuracy, only the translation error is considered, and thus the Average Translation Error is defined as follows:

(25) $$ATE_{trans}=\sqrt{\frac{1}{N}\sum_{i=1}^{N}{\left\|T_{trans}(T_{gt,i}^{-1}T_{esti,i})\right\|}_{2}^{2}}.$$

In the above equation, $T_{gt,i}$ and $T_{esti,i}$ represent the true and estimated values, respectively. $T_{trans}$ denotes the translation portion of the variable inside the parentheses. The RMSE values in Table 1 and Table 2 were obtained using an EVO (Evaluation of Odometry, version 1.29.0) tool. The VEOS3-TEDM algorithm was evaluated using two datasets: one from a long straight corridor scene and another from a laboratory scene. The frame rate of the camera was 15 Hz, while the IMU and wheel encoders operated at 200 Hz. The corridor dataset contains 897 frames, and the laboratory dataset contains 875 frames. Table 1 shows the results of the algorithm comparison for the corridor dataset, while Table 2 presents the results for the laboratory dataset. An “X” in the table indicates that the value could not be computed, meaning that the algorithm failed to track or complete initialization. The pose estimation ratio is defined as the number of frames successfully tracked by the algorithm divided by the total number of frames in the dataset.

The texture of the corridor in those datasets is very weak. During the turn of the intelligent vehicle, the camera is very close to the white wall surface, resulting in a very small number of feature points or even none at all. This causes purely visual SLAM tracking to fail, so both the ORB-SLAM2 and ORB-SLAM3 algorithms using the RGB-D camera fail to track. In Table 1, it can be observed that two types of algorithms are unable to compute the RMSE and the average tracking time per frame. However, ORB-SLAM3, with its multiple map modes, achieves a final pose estimation ratio that is twice as high as that of the ORB-SLAM2 algorithm. When ORB-SLAM3 utilizes the combined form of RGB-D and IMU, the IMU pre-initialization requires the intelligent vehicle to perform sufficient motion to achieve the necessary excitation for IMU initialization. During this time, visual tracking must not fail for an extended period. Nevertheless, visual tracking tends to fail during the vehicle’s turning process. The IMU was unable to complete initialization within the dataset. While the intelligent vehicle’s pose estimation can be achieved using only the wheel encoder, this method results in the shortest average tracking time per frame but produces a relatively high RMSE, which does not meet practical requirements. As shown in Table 1, the VEOS3-TEDM algorithm proposed in this paper outperforms the other algorithms in the corridor datasets. Specifically, in terms of RMSE, it achieves reductions of 38 cm, 1.1 cm, 3.1 cm, and 2.4 cm compared to the encoders and VEORB-SLAM3, VIEORB-SLAM3, and VIEOS3-TEDM algorithms, respectively. Regarding average tracking time per frame, the VEOS3-TEDM algorithm is 3 ms, 12 ms, and 9 ms faster than the VEORB-SLAM3, VIEORB-SLAM3, and VIEOS3-TEDM algorithms, respectively. These results indicate that the VEOS3-TEDM algorithm offers superior performance in both RMSE and average tracking time per frame within the corridor dataset.

The laboratory dataset is rich in texture information, but the high-speed motion of the intelligent vehicle causes tracking failures in ORB-SLAM2. After adding the IMU, ORB-SLAM3 has more stringent initialization requirements, which the intelligent vehicle platform struggles to meet. This results in the inability to calculate the RMSE and average tracking time per frame. However, the final pose estimation ratios of ORB-SLAM3 and ORB-SLAM2 are nearly identical in these datasets. Table 2 shows that, in the laboratory dataset, the VEOS3-TEDM algorithm proposed in this paper achieves RMSE reductions of 29.5 cm, 2.97 cm, 2.34 cm, 4.1 cm, 1.84 cm, and 2.8 cm compared to the encoders and ORB-SLAM3, VEORB-SLAM3, VIEORB-SLAM3, VOS3-TEDM, and VIEOS3-TEDM algorithms, respectively. In terms of average tracking time per frame, VEOS3-TEDM is 6 ms, 5 ms, 17 ms, 4 ms, and 13 ms faster than ORB-SLAM3, VEORB-SLAM3, VIEORB-SLAM3, VOS3-TEDM, and VIEOS3-TEDM, respectively. These results imply that the VEOS3-TEDM algorithm outperforms other algorithms in both RMSE and average tracking time per frame in the laboratory dataset.

Table 1 and Table 2 show that the ORB-SLAM3 algorithm fails to track both in the corridor and laboratory datasets when implemented using RGB-D and IMU fusion. This failure is attributed to the lack of feature points, fast motion, and light reflections. However, the VEOS3-TEDM algorithm proposed in this paper effectively addresses these temporary failure issues, achieving satisfactory accuracy and successfully recovering the entire trajectory. The results demonstrate that the VEOS3-TEDM algorithm generally achieves better accuracy and stability due to the fusion of ORB triangulation estimation with RGB-D depth measurement and the integration of encoder information. This approach aids in identifying outliers, enhancing prediction, and improving pose estimation. The improvements are particularly noticeable in datasets with higher motion speeds.

**Figure 4:**
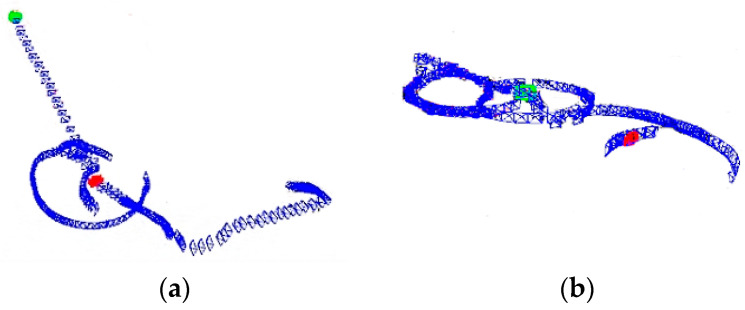
The process of running datasets in the VEOS3-TEDM algorithm: (**a**) corridor scene and (**b**) laboratory scene. The blue frames represent keyframes, red frames represent initial keyframes, and green frames represent current frames.

The process of running the VEOS3-TEDM algorithm on the corridor and laboratory datasets is illustrated in Figure 4. During the execution, the pose of all keyframes is based on the world coordinate system, which is established at the frame where initialization is completed. After initialization, map points are created and their 3D coordinates are relative to the world coordinate system. The map points are categorized into two types: local map points and global map points. Local map points assist in localization during the tracking process. In Figure 4, the blue frames represent keyframes, red frames represent initial keyframes, and green frames represent current frames. The VEOS3-TEDM algorithm achieves satisfactory accuracy and successfully recovers the entire trajectory. This demonstrates that the VEOS3-TEDM algorithm, which integrates ORB triangulation estimation, RGB-D depth measurement, and encoder information, effectively fulfills the primary goal of this paper.

**Figure 5:**
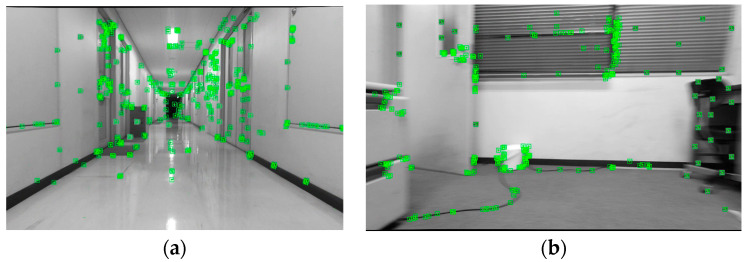
The process of tracking datasets in the VEOS3-TEDM algorithm: (**a**) corridor scene and (**b**) laboratory scene. The green boxes in the figure represent key feature points detected by VEOS3-TEDM algorithm.

The tracking process of the VEOS3-TEDM algorithm on the corridor and laboratory datasets is shown in Figure 5, where the green boxes indicate the recognized 3D feature points. In Figure 5, it is evident that angular feature points and those with significant changes in gray value are well identified. This demonstrates that the VEOS3-TEDM algorithm can stably track the image without failure. This stability is achieved by incorporating encoder information into the algorithm. Even if the visual tracking fails, the encoder data can compensate, allowing the algorithm to connect the two states and maintain continuous stable tracking.

**Figure 6:**
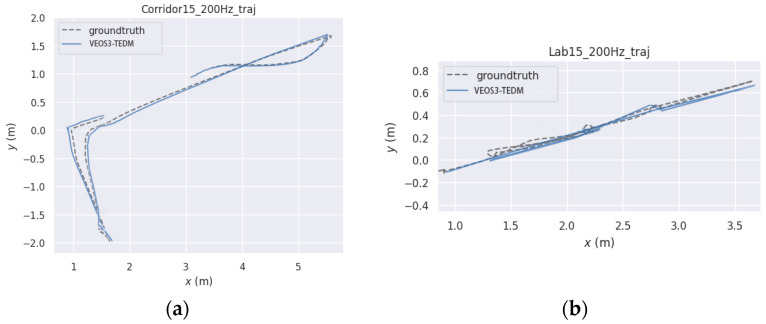
The comparison between estimated and true trajectory in the VEOS3-TEDM algorithm: (**a**) corridor scene and (**b**) laboratory scene.

When the VEOS3-TEDM algorithm finishes running, it generates the pose information of the keyframes. Figure 6 shows a comparison between the pose estimates generated by the VEOS3-TEDM algorithm using the open-source software EVO and the true trajectories. As seen in Figure 6, the estimated trajectories closely match the true trajectories on flat and straight roads, with only minor errors observed in the corridor scene. However, there is some deviation in the vehicle’s trajectory at turns, where the camera is very close to the white wall, resulting in few or no feature points. This causes the pure vision ORB-SLAM3 tracking to fail. Nonetheless, the VEOS3-TEDM algorithm is able to track continuously and stably due to the compensation provided by encoder data. In the laboratory scene, the estimated trajectory of the vehicle closely matches the true trajectory, as the scene is rich in texture information, enabling accurate recognition of 3D feature points, and thus producing a more accurate estimated trajectory.

**Figure 7:**
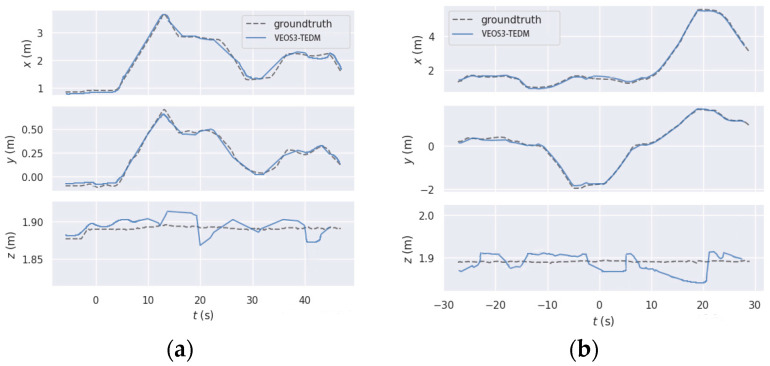
The comparison between true and estimated trajectories in *x*, *y* and *z* directions, using the VEOS3-TEDM algorithm: (**a**) corridor scene and (**b**) laboratory scene.

As shown in Figure 7, the trajectory estimation errors in the *x*-axis and *y*-axis directions are relatively small in both the corridor and laboratory scenes. However, the errors in the *z*-axis direction are comparatively larger. This is due to the fact that encoder information is recorded more accurately in the horizontal direction, while larger errors are observed in the vertical direction. This suggests that further improvements are needed in the algorithms to enhance the accuracy of depth value processing. Additionally, the trajectory estimation errors in the *x*-axis and *y*-axis directions are smaller in the corridor scene compared to the laboratory scene. This is likely because the corridor scene is more open, with fewer obstacles, leading to a more accurate trajectory estimation in the horizontal directions.

**Figure 8:**
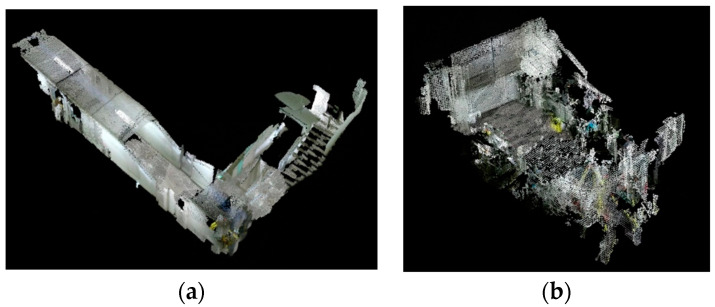
3D point cloud maps: (**a**) corridor scene and (**b**) laboratory scene.

Finally, the intelligent vehicle successfully constructed 3D point cloud maps of the corridor and laboratory scenes, as shown in Figure 8. The constructed 3D point cloud maps are consistent with the actual scenes. For approximate navigation, the positional accuracy of obstacles, which are defined as points located between the ground plane and the height of the robot, is considered acceptable. However, some redundancies remain due to inevitable errors introduced by the pure encoders, which can be minimized through more closed-loop detection, image measurements, and better prior calibration. The point cloud representation of the map points is mainly used to highlight visible errors in the estimated trajectories of the keyframes. However, for ROS navigation, future research should focus on using a grid map form that incorporates advanced filtering techniques [43].

### 5.2. Experimental Analysis of Real-World Environments

**Figure 9:**
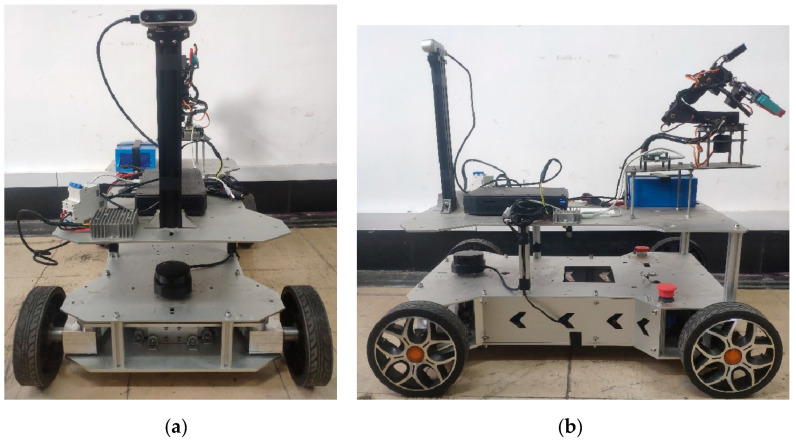
Images of the experimental platform: (**a**) front view and (**b**) left view.

**Figure 10:**
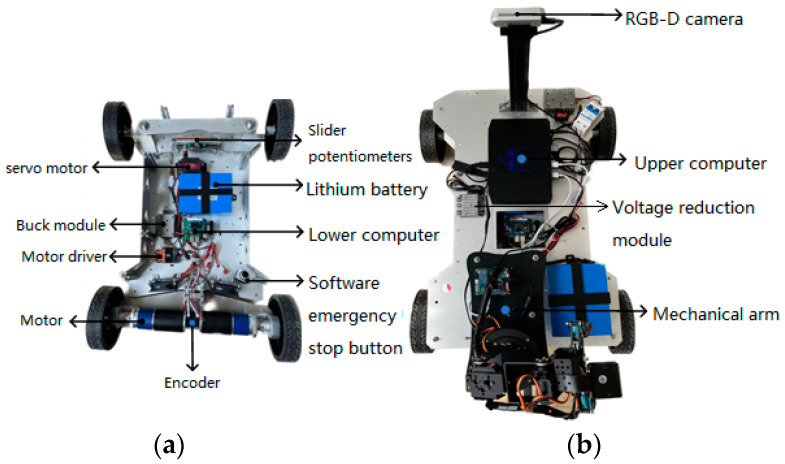
The location of various components on the mobile robot: (**a**) bottom level and (**b**) upper level.

The experimental platform is shown in Figure 9. The mobile robot is divided into a bottom layer, an intermediate layer, and an upper layer. The bottom layer contains the lithium battery, lower computer, motor driver, software-based emergency stop button, servo motor, and motor. The middle layer houses the single-wire LiDAR, power switch, and emergency stop button. The upper layer contains the upper computer, RGB-D camera, mechanical arm, voltage reduction module, and power switch for the upper computer. The locations of the devices held in the bottom and upper layers of the mobile robot are shown in Figure 10.

The proposed VEOS3-TEDM algorithm is first ported to a mobile robot. Afterward, the algorithm is validated using the mobile robot. In this paper, data sequences of real-world indoor scenes are captured by an experimental platform. For the collected indoor scene data, it is challenging to obtain the ground truth for each moment on the experimental platform. Instead, the dataset is collected by controlling the motion of the experimental platform so that the data sequence forms a large closed loop at the end. Specifically, the experimental platform starts at the initial point, completes a full circle, and returns to the starting point. Thus, the start and end segments of the data sequence are in the same scenario.

**Figure 11:**
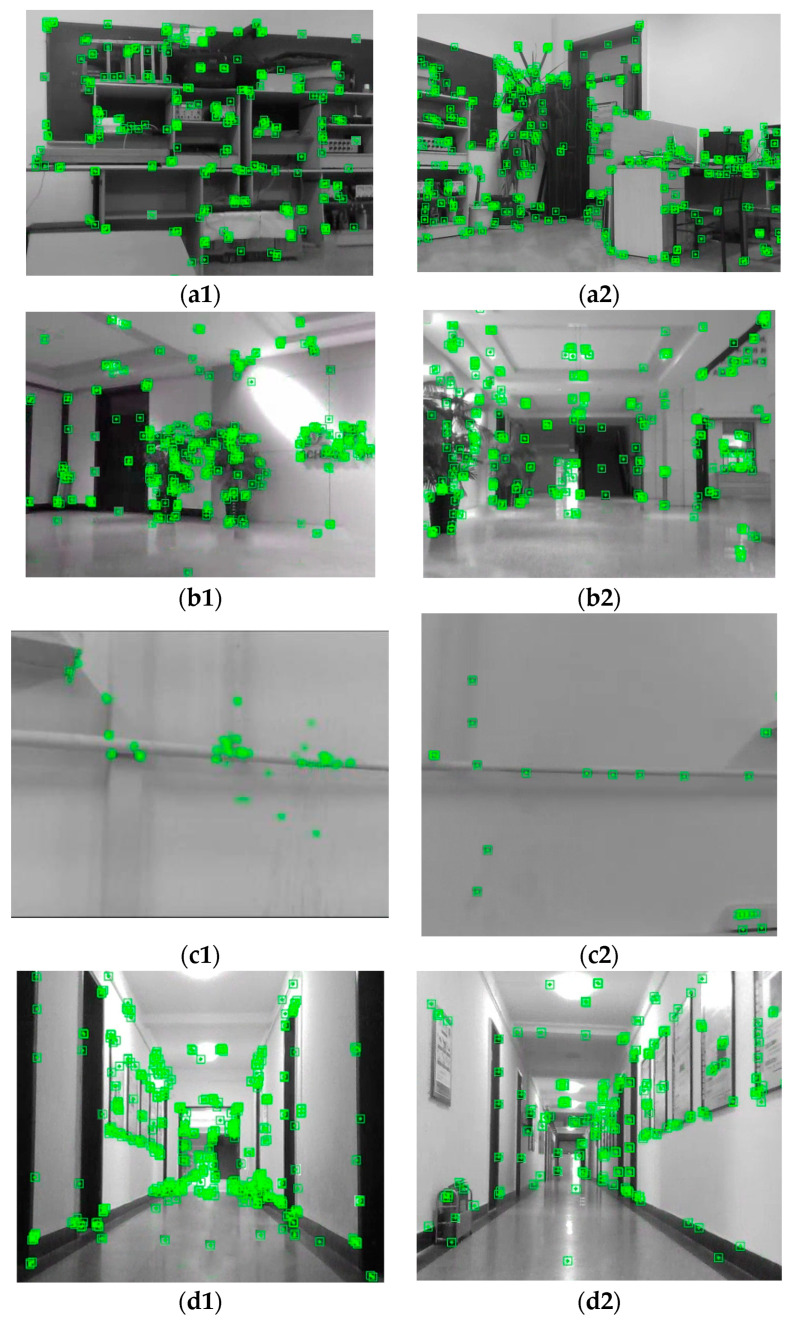
The process of tracking real-world environments in the VEOS3-TEDM algorithm: (**a1**,**a2**) laboratory, (**b1**,**b2**) hall, (**c1**,**c2**) weak texture scene, (**d1**,**d2**) long straight corridor. The green boxes in the figure represent key feature points detected by VEOS3-TEDM algorithm.

The RGB-D camera used in the process has a frame rate of 30 Hz and an image resolution of 640 × 480. The IMU operates at 200 Hz, and the wheel encoders operate at 100 Hz. The data from the wheel encoders are collected by the lower computer and transmitted to the upper computer via serial communication. The operating environments include the laboratory, a long straight corridor, and a hall. These environments are relatively large, and the tracking process of the experimental platform during the experiment is shown in Figure 11. Figure 11 indicates that the experimental platform captures a relatively large number of 3D feature points in the laboratory, corridor, and hall scenes, including distinct angular points and points with significant changes in gray value. This demonstrates that the VEOS3-TEDM algorithm achieves the desired accuracy and stability. However, the 3D feature points obtained in weakly textured scenes (e.g., white walls) are relatively sparse, suggesting that texture richness directly impacts the accuracy and stability of localization and map building. Therefore, to achieve high localization and map-building accuracy, the mobile robot should operate in texture-rich scenes.

**Figure 12:**
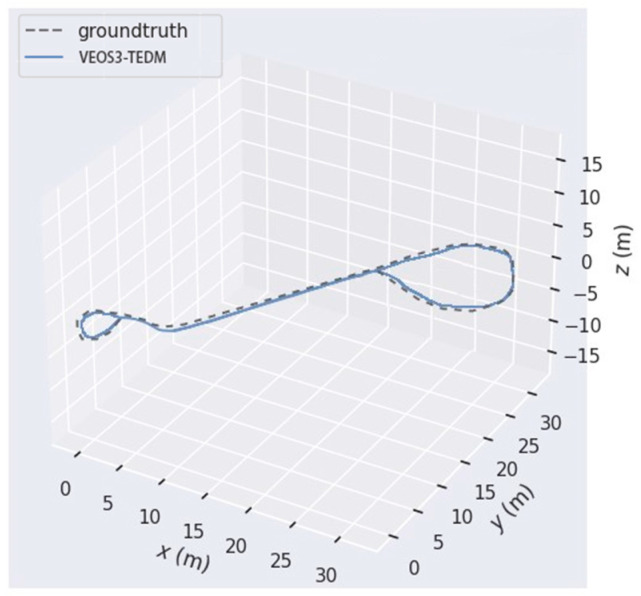
A comparison of estimated and true trajectories in real-world environments using the VEOS3-TEDM algorithm.

Figure 12 illustrates the estimated trajectory of VEOS3-TEDM running in a real-world environment compared to the ground truth, with an obtained RMSE of 0.091. The effectiveness and robustness of the proposed VEOS3-TEDM algorithm were validated in real-world environments. Additionally, the estimated trajectory closely aligns with the ground truth, achieving the research objectives outlined in this paper.

## 6. Conclusions

This paper introduces wheel odometry into the classical ORB-SLAM3 framework. The main contribution is the proposal of a novel combination of CI-TEDM and wheel odometry, offering a new approach to enhancing the accuracy and robustness of SLAM. Additionally, this paper derives the wheel encoder pre-integration model, error model, rotation-to-state-variable Jacobian matrix, and position-to-state-variable Jacobian matrix, all of which are employed for local BA optimization to improve SLAM results. Through these advancements, a more accurate and robust VEOS3-TEDM algorithm is achieved. Experiments on open-source datasets and in real-world environments demonstrate that the proposed VEOS3-TEDM algorithm surpasses state-of-the-art methods in both positioning and mapping accuracy. Furthermore, the proposed method is extensible to other optimization-based indirect visual SLAM or visual-inertial SLAM systems.

## Figures and Tables

**Table 1:** The comparison of algorithm results on the corridor dataset.

Algorithm Name	Sensors	RMSE (m)	Average Tracking Time per Frame (ms)	Pose Estimation Ratio (%)
	Encoders	0.463	1	100
ORB-SLAM2	RGB-D	X	X	45
ORB-SLAM3	RGB-D	X	X	94
	RGB-D + IMU	X	X	5
VEORB-SLAM3	RGB-D + Encoders	0.094	17	100
VIEORB-SLAM3	RGB-D + IMU + Encoder	s0.114	26	100
VOS3-TEDM	CI-TEDM	X	X	96
VEOS3-TEDM	CI-TEDM + Encoders	0.083	14	100
VIEOS3-TEDM	CI-TEDM + IMU + Encoders	0.107	23	100

**Table 2:** The comparison of algorithm results on the laboratory dataset.

Algorithm Name	Sensors	RMSE (m)	Average Tracking Time per Frame (ms)	Pose Estimation Ratio (%)
	Encoders	0.382	1	100
ORB-SLAM2	RGB-D	X	X	92
ORB-SLAM3	RGB-D	0.1167	22	100
	RGB-D + IMU	X	X	5
VEORB-SLAM3	RGB-D + Encoders	0.1104	21	100
VIEORB-SLAM3	RGB-D + IMU + Encoders	0.128	33	100
VOS3-TEDM	CI-TEDM	0.1054	20	100
VEOS3-TEDM	CI-TEDM + Encoders	0.087	16	100
VIEOS3-TEDM	CI-TEDM + IMU + Encoders	0.115	29	100

## Data Availability

Data are contained within the article.
